# Effect of Mo Addition on the Chemical Corrosion Process of SiMo Cast Iron

**DOI:** 10.3390/ma13071745

**Published:** 2020-04-09

**Authors:** Marcin Stawarz, Paweł M. Nuckowski

**Affiliations:** 1 Department of Foundry Engineering, Silesian University of Technology, 7 Towarowa Street, 44-100 Gliwice, Poland; 2 Department of Engineering Materials and Biomaterials, Silesian University of Technology, 18A Konarskiego Street, 44-100 Gliwice, Poland; pawel.nuckowski@polsl.pl

**Keywords:** chemical corrosion, SiMo cast iron, fayalite, hematite, magnetite, maghemite, sulfur dioxide

## Abstract

The study was carried out to evaluate five SiMo cast iron grades and their resistance to chemical corrosion at elevated temperature. Corrosion tests were carried out under conditions of an actual cyclic operation of a retort coal-fired boiler. The duration of the study was 3840 h. The range of temperature changes during one cycle was in the range of 300–650 °C. Samples of SiMo cast iron with Si content at the level of 5% and variable Mo content in the range 0%–2.5% were used as the material for the study. The examined material was subjected to preliminary metallographic analysis using scanning microscopy and an Energy dispersive spectroscopy (EDS) system. The chemical composition was determined on the basis of a Leco spectrometer and a Leco carbon and sulfur analyzer. The examination of the oxide layer was carried out with the use of Scanning electron microscope (SEM), EDS, and X-ray diffraction (XRD) methods. It was discovered that, in the analyzed alloys, oxide layers consisting of Fe_2_O_3_, Fe_3_O_4_, SO_2_, and Fe_2_SiO_4_ were formed. The analyzed oxide layers were characterized by high adhesion to the substrate material, and their total thickness was about 20 μm.

## 1. Introduction

Corrosive behavior of SiMo cast iron in the air and flue gases was presented in the works [1,2,3,4,5]. When we subject pure iron to the corrosion process at elevated temperatures and in the ambient atmospheric air, a multilayer oxide structure composed of FeO, Fe_3_O_4_, and Fe_2_O_3_ is formed [5]. For ductile cast iron, an oxide layer is formed, located both in the material and in the surface layer as a result of migration of Fe atoms [5]. After introducing an alloying element in the form of Si into cast iron, a SiO_2_ compound is formed at the metal–oxide layer point of contact, which constitutes a barrier to further oxidation processes [5]. Simultaneously, SiO_2_ can react with O, Fe, and FeO. The result of these reactions may be the formation of fayalite, Fe_2_SiO_4_ [6,7,8]. In the paper [9], the author writes that the oxide layer on the surface of SiMo cast iron is composed of the following sub-layers situated from the outside to the inside of the material: Fe_2_O_3_, Fe_3_O_4_, FeO, FeO + Fe_2_SiO_4_.

The oxide layer adheres well to the base material and the inner layer consisting of FeO + Fe_2_SiO_4_ [9]. The higher the silicon content in the base material, the faster the oxide layer forms. A number of studies concerning the corrosion resistance of SiMo cast iron focus on a relatively short time of exposure to oxidation (500 h on average) [10]. These studies are conducted mainly in terms of the use of SiMo cast iron in automotive castings, as described by Rouczka [11] and many other authors [12,13,14,15,16,17]. SiMo cast iron is an increasingly popular material, and research on this material is also conducted with a focus on optimizing the manufacturing process. In their work, Guzik et al. [18] write about the method of introducing two flexible hoses with the diameter of Ø 9 mm; one filled with a FeSi + Mg mixture, and the other with a graphitizing modifier for the treatment drum ladle. Guzik et al. [18] describe it as a new method of secondary treatment of ferritic cast iron production of SiMo type. This method can be used for the production of ductile iron melted in an induction furnace [18,19].

SiMo cast iron can also be successfully used in other areas of industry: exhaust parts for combustion engines, turbocharger housings and rotors, gas turbine components, molds for the glass industry, molds for aluminum alloys, zinc, forging dies, heat treatment furnace components, aluminum melting furnace components, and waste incineration furnaces. This happens wherever elevated operating temperatures and gases resulting from the combustion, e.g., of solid fuels are involved. A good example of such a system is a coal-fired retort furnace. Nyashina and her team write about the problems related to the emission of pollutants during the combustion process [20]. Released into the atmosphere with exhaust gases, nitrogen oxides (mainly NO and NO_2_) are the main reason why photochemical smog appears, which reaches the stratosphere to act as a catalyst for ozone layer depletion. Rapid oxidation of NOx and SOx and their interaction with water vapor in the atmosphere generates tiny droplets of sulfuric (H_2_SO_4_) and nitric (HNO_3_) acids [20]. Sulfuric acid causes significant losses in the ecosystem, which has been mentioned by many authors [21]. It is important to optimize the combustion process of solid fuels in boilers by improving the materials from which these boilers are built. For the above reasons, in this work, studies of resistance to chemical corrosion of SiMo cast iron were carried out during actual operation of a retort boiler. The duration of the study was 3840 hours. To date, the corrosion resistance of SiMo cast iron during the operation of a retort boiler has not been described in the literature. Due to its properties, it can be successfully used for manufacturing furnace elements fired with solid, liquid, or gaseous fuels.

## 2. Methods and Materials

**Figure 1:**
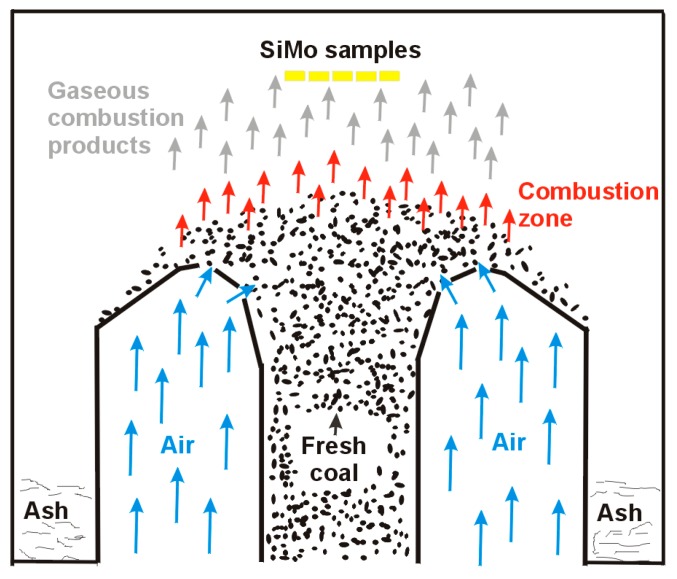
Scheme of a single retort with (yellow) SiMo samples placed above the stoker.

Experimental melts were conducted in an induction furnace (PI25, ELKON Sp. z o.o., Rybnik, Poland) with medium frequency and a capacity of 25 kg. The charge consisted of steel scrap with low sulphur content. Other ingredients added during the melting were ferrosilicon FeSi75, synthetic graphite of carbon content above 99.35%, and FeMo65-rich alloy. The spheroidization process of cast iron was conducted at the bottom of the ladle after covering the nodulizing agent with pieces of steel scrap. The magnesium-rich alloy used in the studies was FeSiMg5RE. The studies were carried out under conditions of cyclic temperature changes in the range of 300–650 °C. The duration of the study was 3840 h. The full cycle time of heating and cooling was 12 min 30 s. A total of 18,432 full cycles of heat load were carried out. The length of the test cycle was selected so that the samples would reach the assumed minimum and maximum temperatures. Temperature measurement of the samples was performed with a NiCr-Ni thermocouple, with no recording of temperature changes in time, and the measurement of surface temperature of the samples was performed to determine the minimum and maximum temperature for the test cycle. The tests were carried out under conditions of a reverberatory furnace (the scheme of a single retort stoker is shown in Figure 1).

The fuel used was bituminous coal with a calorific value of 26–28 MJ/kg, a combustion heat of 29 MJ/kg, a granulation of 5–25 mm, a humidity of <10%, a maximum ash content of 7%, and a maximum sulfur content of 0.6%. The fuel used was certified by Główny Instytut Górnictwa (Central Mining Institute).

In the studies, samples of SiMo cast iron with Si content of 5% and Mo content of 0%–2.5% were used. The chemical composition was determined on the basis of a Leco spectrometer (Model No 607-500, Leco Corporation, 3000 Lakeview Ave, St. Joseph, MI, USA) and a CS-125 Leco carbon and sulfur analyzer (Leco Corporation, 3000 Lakeview Ave, St. Joseph, MI, USA). The chemical composition of the tested samples is presented in Table 1.

In order to determine the phase composition of the studied material, X-ray diffraction analyses were carried out with the use of an X’Pert Pro multipurpose x-ray diffractometer by Panalytical (Almelo, The Netherlands). The measurements were conducted utilizing filtered radiation of a cobalt anode lamp (λKα = 0.179 nm) as well as a PIXcel 3D detector on the diffracted beam axis. The diffraction lines were recorded in the Bragg–Brentano geometry in the angular scope of 10°–120° (2θ), with the step of 0.026° and the step time of 100 s. Furthermore, to obtain more precise information from the surface oxide layer, grazing incidence diffraction (GID) geometry with a proportional detector on the diffracted beam axis was used. In this geometry, a primary X-ray beam was set at a constant, low angle (1.5^o^) related to the sample plane, which affected the corresponding slope of diffraction vectors related to the normal to surface. This allowed us to obtain during the measurement a constant penetration depth of the X-ray beam, limited mainly to the surface oxide layer. The analysis of the obtained diffraction patterns was made in the Panalytical High Score Plus software (ver.: 3.0e), with the dedicated Panalytical Inorganic Crystal Structure Database (PAN-ICSD).

The analysis of the structure and the chemical composition was performed on a Phenom ProX scanning microscope (Phenom-World Eindhoven, Noord-Brabant, Netherlands) equipped with an energy-dispersive X-ray spectrometer (EDS).

## 3. Results

### 3.1. SEM Analysis

**Figure 2:**
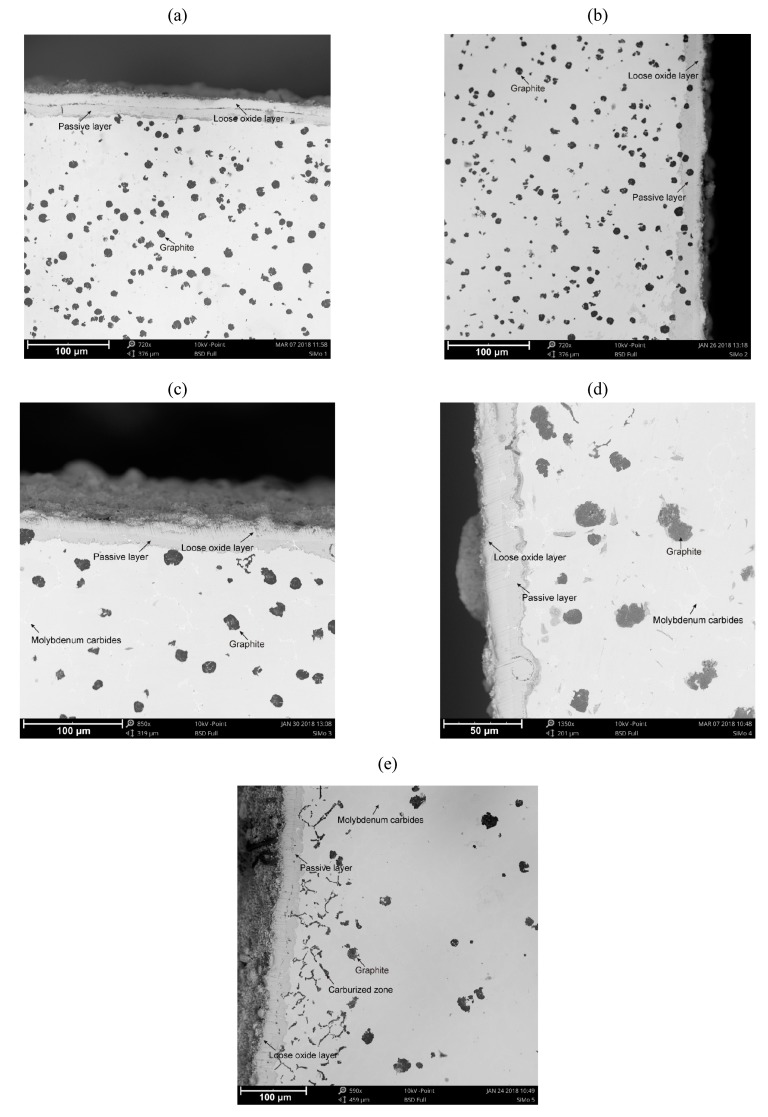
Microstructure of SiMo cast iron after the corrosion test cycle. SiMo cast iron with addition of: (**a**) 0.01% Mo, (**b**) 0.47% Mo, (**c**) 1.09% Mo, (**d**) 1.92% Mo, (**e**) 2.51% Mo. SEM.

Figure 2 shows photos of SiMo cast iron samples after the chemical corrosion resistance test cycle. The microstructure of the cast iron consisted of a ferritic matrix, graphite nodules, molybdenum carbide (Mo_2_C), a carburized zone (Figure 2e), and the passive layer and the loose oxide layer on the top surface of the samples. Microstructure components are highlighted in Figure 2.

In all cases (SiMo samples 1–5), the thickness of passive layers is around 10 μm. For elevated molybdenum content (Figure 2e, SiMo melt 5, 2.51% Mo) in the near-surface layer, the carburized zone in the form of black inclusions distributed in the vicinity of Mo_2_C carbide precipitates is clearly visible. All the cases considered are characterized by a cohesive passive layer tightly adhering to the sample. No cracks nor defects in the passive layer were observed, even after the process of preparing metallographic sections (cutting, grinding, and polishing).

### 3.2. EDS Analysis

**Figure 3:**
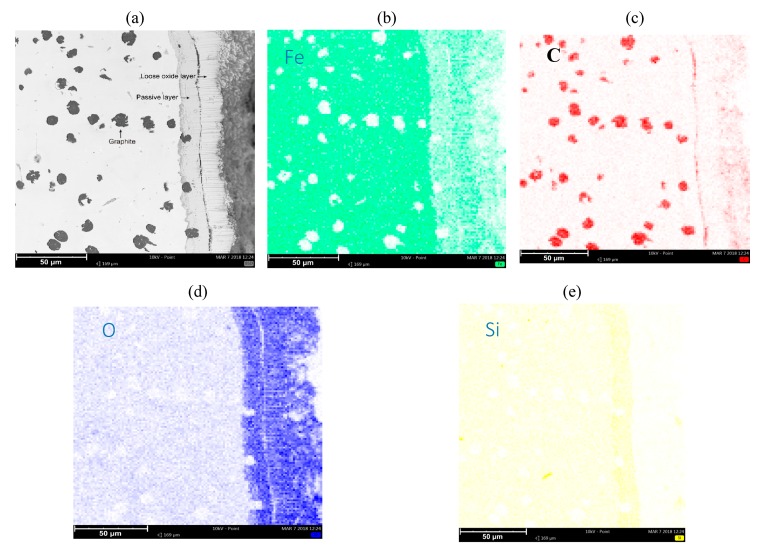
Microstructure of SiMo cast iron, 0.01% Mo (**a**). Elements decomposition maps. Map for (**b**) Fe, (**c**) C, (**d**) O, and (**e**) Si.

**Figure 4:**
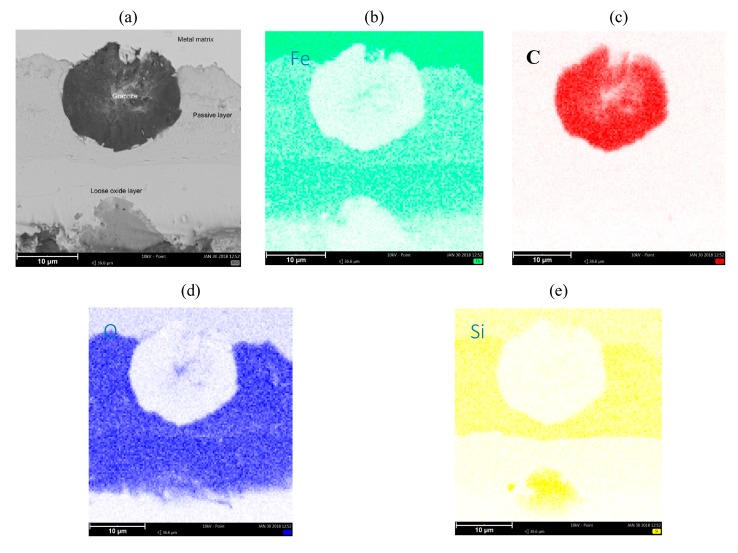
Microstructure of SiMo 3 cast iron, 1.09% Mo (**a**). Map for (**b**) Fe, (**c**) C, (**d**) O, and (**e**) Si.

**Figure 5:**
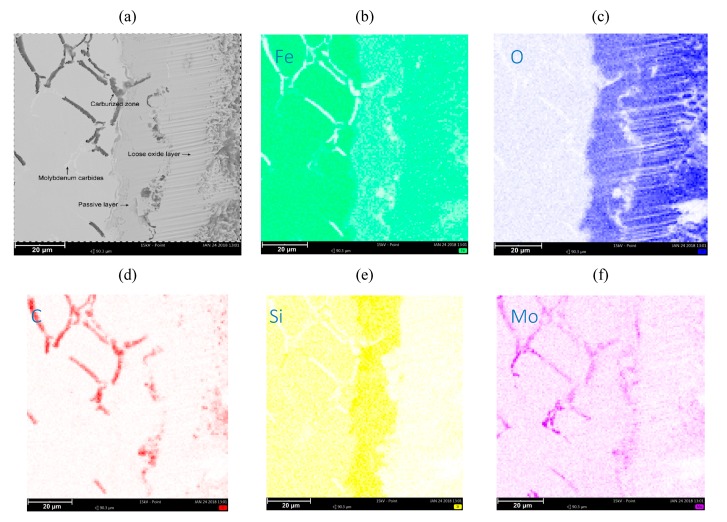
View of the carburized layer. SiMo sample 5. 2.51% Mo (**a**). Map for (**b**) Fe, (**c**) O, (**d**) C, (**e**) Si, and (**f**) Mo.

Figure 3 and Figure 4 show the collective results of metallographic studies for selected alloys using the EDS system. The presented results indicate the presence of a loose oxide layer (blue), which is adjacent to the passive layer. The passive layer is an area also marked in blue, where the area has an increased silicon content (intense yellow bands on the maps—see Figure 3e, Figure 4e, Figure 5e). The increased Si content in the passive layer results from the diffusion of iron atoms from this layer to the loose oxide layer, which makes the area richer in Si. Penetrating oxygen reaches the area enriched with silicon and forms compounds with it (e.g., SiO_2_) creating a tight barrier to the propagation of corrosive phenomena. Of course, a SiO_2_ compound can react with Fe, O, and FeO, forming a fayalite Fe_2_SiO_4_ [8,17].

The characteristic phenomenon of these alloys is their ability to perform so-called “self-healing”. Removing the passive layer creates a new one in its place. The average thickness of the loose oxide layer was 10 μm for the tested samples, while the thickness of the passive layer was also around 10 μm. Of course, the thickness of this layer depends on the local conditions on the surface of the sample exposed to the corrosive agents.

Figure 5 shows the analysis of the sample area (for the SiMo melt 5), where a significant degree of carburation of the casting material layer was observed. The layer is placed under the passive layer. This can be explained by the high concentration of carbon in the corrosive atmosphere of the furnace, and the penetration of carbon atoms through the passive layer towards the center of the casting.

### 3.3. XRD Analysis

**Figure 6:**
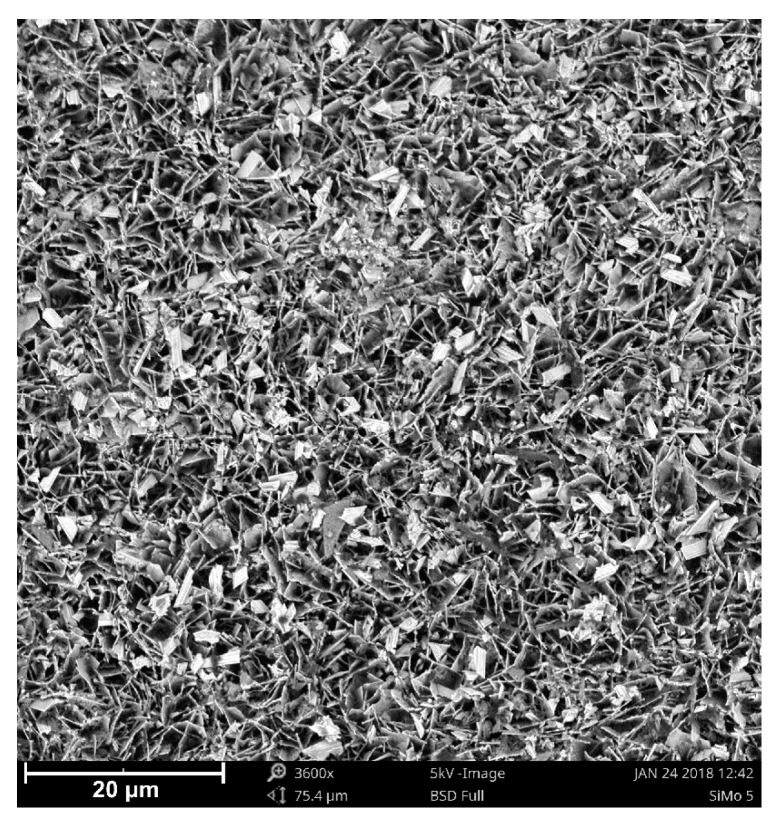
View of the loose oxide layer on a SiMo 5 sample. SEM.

**Figure 7:**
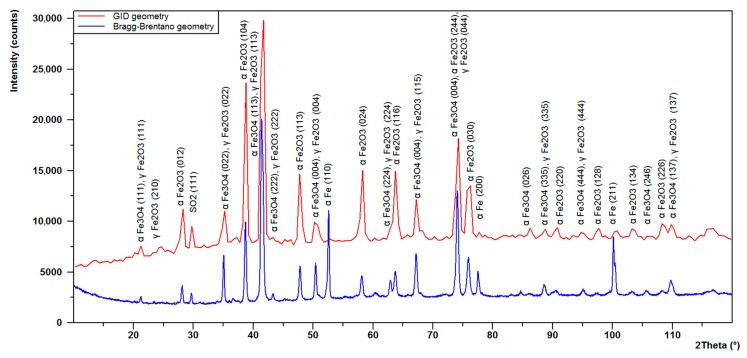
Diffractogram from a sample. Blue chart, Bragg–Brentano geometry; red chart, grazing incidence diffraction (GID) geometry. SiMo 3. 1.09% Mo.

The above SEM and EDS results were complemented with X-ray diffraction. Two measurements of the surface area of a selected sample were made (Figure 6), one with Bragg–Brentano geometry and the other one using the grazing incidence diffraction (GID) geometry. The GID geometry allows us to limit the penetration of the X-ray beam, so that diffractive information can be obtained mainly from the surface layer (in this particular case, the evidence is the almost complete disappearance of the diffraction lines from the substrate). In the figure below Figure 7, two diffractograms are superimposed on each other. The blue diffractogram was obtained using Bragg–Brentano geometry and the red diffractogram using GID geometry at a 1.5-degree tilt of the primary beam in relation to the measurement plane.

From the results of the analysis presented in Figure 7, the following components of the surface oxide layer were identified. The compounds identified were, among others: α - Fe_2_O_3_ (hematite) with a hexagonal lattice R-3c (98-002-2505), α - Fe_3_O_4_ (magnetite) with a cubic lattice Fd-3m (98-026-3007), γ - Fe_2_O_3_ (maghemite) with a cubic lattice Fd-3m (98-017-2905), and a small share of SO_2_ (an orthorhombic lattice).

## 4. Discussion

The presented photos of SiMo cast iron samples (Figure 2), after a series of chemical corrosion resistance tests, clearly show the microstructure of the cast iron with the passive layer and the loose oxide layer on the surface of the samples. The obtained results are analogous to the results presented by M. Ekström in his paper [5]. The results obtained by M. Ekström were obtained under different test conditions in a flue gas atmosphere. For all cases analyzed in the study, the thickness of the oxide and passive layers oscillates around the level of 20 μm. For elevated molybdenum content (SiMo melt 5, 2.51% Mo) in the near-surface layer, the carburized zone, in the form of black inclusions distributed in the vicinity of Mo_2_C carbide precipitates, is clearly visible. The layer is placed under the passive layer, which is clearly visible in Figure 5. We found no description of a similar case in the available literature. The obtained effect can be explained by the high concentration of carbon in the corrosive atmosphere of the furnace; penetration of carbon atoms through the passive layer in the direction of the casting center, combined with the high concentration of molybdenum in the casting, resulted in the accumulation of carbon atoms around the precipitates rich in molybdenum (Mo_2_C carbides).

The results obtained from the XRD analysis allowed for the following components of the surface oxide layer to be described, among others: α - Fe_2_O_3_ (hematite), α - Fe_3_O_4_ (magnetite), γ - Fe_2_O_3_ (maghemite), and SO_2_ [22,23,24].

The X-ray diffraction patterns of magnetite and maghemite are very similar. This is related to their similar structures. Both of these two oxide phases crystallize in the cubic system and their lattice parameters are very close. For this reason, it is difficult to differentiate these structures. However, some works [25,26] report that the maghemite phase gives two additional small diffraction lines at 23.77^o^ (210) and 26.10^o^ (211). One of these, line (210), was identified even on a diffractogram obtained in Bragg–Brentano geometry. The formed magnetite and maghemite oxide layers do not show the much higher intensity of line (113) in relation to the highest hematite line (104). This result can be explained by the similar crystal growth of these oxide phases. Also, some authors report that the formation of maghemite (γ - Fe_2_O_3_) is a result of oxidation of the magnetite (α - Fe_3_O_4_) [27,28]. One of the variables deciding the quantitative share of hematite, magnetite, and maghemite is the range of oxidation temperature described by M. Marciuš et al. [29].

## 5. Conclusions

Based on the conducted study, it can be stated that SiMo cast iron is fully resistant to chemical corrosion during retort furnace operations. All SiMo cast iron samples were characterized by a cohesive oxidized layer, consisting of a passive layer on the casting material side and an oxide surface layer.

The two layers adhered quite well to one another. No cracks nor defects in any of the elements of the oxidized layer were observed. The surface oxide layer was found to consist of the following compounds: Fe_2_O_3_, Fe_3_O_4_, and SO_2_.

For the sample with increased Mo content, a significant carburization of the near-surface layer of the sample was observed, especially in the areas adjacent to molybdenum carbide. The carburized edge zone of the sample did not affect the corrosion resistance of SiMo cast iron.

The areas strongly enriched with silicon are the fayalite Fe_2_SiO_4_ resulting from the reaction of SiO_2_ with O, Fe, and FeO.

Due to the relatively low operating temperature range, we suggest that the Mo content in the alloy be reduced to the range of 0%–0.5% Mo.

## Figures and Tables

**Table 1:** Chemical composition of the tested SiMo cast iron.

Melt Number	Chemical Composition, % of Weight						
	C	Si	Mo	P	S	Mg	Fe (Balance)
SiMo 1	3.02	5.03	0.01	0.021	0.009	0.032	91.878
SiMo 2	2.94	5.14	0.47	0.020	0.005	0.029	91.396
SiMo 3	3.04	4.94	1.09	0.022	0.005	0.031	90.872
SiMo 4	2.71	5.17	1.92	0.018	0.009	0.062	90.111
SiMo 5	2.74	4.42	2.51	0.022	0.007	0.033	90.268
